# Supplementary material for: Iterative improvement in the automatic modular design of robot swarms
Source: PeerJ Comput Sci. 2020 Dec 7;6:e322. doi: 10.7717/peerj-cs.322 (PMC7924708; doi:10.7717/peerj-cs.322)
Supplement: Supplemental Information 3 [file peerj-cs-06-322-s003.zip › argos3/doc/api/standalone/a00436_source.html]

ARGoS: plugins/robots/foot-bot/control\_interface/ci\_footbot\_distance\_scanner\_actuator.cpp Source File


- Main Page
- Related Pages
- Namespaces
- Classes
- Files

- File List
- File Members

# plugins/robots/foot-bot/control\_interface/ci\_footbot\_distance\_scanner\_actuator.cpp

Go to the documentation of this file.

```
00001 
00007 #include "ci_footbot_distance_scanner_actuator.h"
00008 
00009 #ifdef ARGOS_WITH_LUA
00010 #include <argos3/core/wrappers/lua/lua_utility.h>
00011 #endif
00012 
00013 namespace argos {
00014 
00015    /****************************************/
00016    /****************************************/
00017 
00018 #ifdef ARGOS_WITH_LUA
00019    int LuaDistanceScannerEnable(lua_State* pt_lua_state) {
00020       /* Check parameters */
00021       if(lua_gettop(pt_lua_state) != 0) {
00022          return luaL_error(pt_lua_state, "robot.distance_scanner.enable() expects no arguments");
00023       }
00024       /* Perform action */
00025       CLuaUtility::GetDeviceInstance<CCI_FootBotDistanceScannerActuator>(pt_lua_state, "distance_scanner")->Enable();
00026       return 0;
00027    }
00028 
00029    int LuaDistanceScannerDisable(lua_State* pt_lua_state) {
00030       /* Check parameters */
00031       if(lua_gettop(pt_lua_state) != 0) {
00032          return luaL_error(pt_lua_state, "robot.distance_scanner.disable() expects no arguments");
00033       }
00034       /* Perform action */
00035       CLuaUtility::GetDeviceInstance<CCI_FootBotDistanceScannerActuator>(pt_lua_state, "distance_scanner")->Disable();
00036       return 0;
00037    }
00038 
00039    int LuaDistanceScannerSetAngle(lua_State* pt_lua_state) {
00040       /* Check parameters */
00041       if(lua_gettop(pt_lua_state) != 1) {
00042          return luaL_error(pt_lua_state, "robot.distance_scanner.set_angle() expects 1 argument");
00043       }
00044       luaL_checktype(pt_lua_state, 1, LUA_TNUMBER);
00045       /* Perform action */
00046       CLuaUtility::GetDeviceInstance<CCI_FootBotDistanceScannerActuator>(pt_lua_state, "distance_scanner")->SetAngle(CRadians(lua_tonumber(pt_lua_state, 1)));
00047       return 0;
00048    }
00049 
00050    int LuaDistanceScannerSetRPM(lua_State* pt_lua_state) {
00051       /* Check parameters */
00052       if(lua_gettop(pt_lua_state) != 1) {
00053          return luaL_error(pt_lua_state, "robot.distance_scanner.set_rpm() expects 1 argument");
00054       }
00055       luaL_checktype(pt_lua_state, 1, LUA_TNUMBER);
00056       /* Perform action */
00057       CLuaUtility::GetDeviceInstance<CCI_FootBotDistanceScannerActuator>(pt_lua_state, "distance_scanner")->SetRPM(lua_tonumber(pt_lua_state, 1));
00058       return 0;
00059    }
00060 #endif
00061 
00062    /****************************************/
00063    /****************************************/
00064 
00065 #ifdef ARGOS_WITH_LUA
00066    void CCI_FootBotDistanceScannerActuator::CreateLuaState(lua_State* pt_lua_state) {
00067       CLuaUtility::OpenRobotStateTable (pt_lua_state, "distance_scanner"                            );
00068       CLuaUtility::AddToTable          (pt_lua_state, "_instance",       this                       );
00069       CLuaUtility::AddToTable          (pt_lua_state, "enable",          &LuaDistanceScannerEnable  );
00070       CLuaUtility::AddToTable          (pt_lua_state, "disable",         &LuaDistanceScannerDisable );
00071       CLuaUtility::AddToTable          (pt_lua_state, "set_angle",       &LuaDistanceScannerSetAngle);
00072       CLuaUtility::AddToTable          (pt_lua_state, "set_rpm",         &LuaDistanceScannerSetRPM  );
00073       CLuaUtility::CloseRobotStateTable(pt_lua_state                                                );
00074    }
00075 #endif
00076 
00077    /****************************************/
00078    /****************************************/
00079 
00080 }
```

---

Generated on 10 Jul 2018 for ARGoS by 
 1.6.1
